# Hybrid rotational atherectomy and shockwave-assisted complex left main PCI with mechanical support in a high-bleeding risk patient, a rare case report

**DOI:** 10.1016/j.ijscr.2025.111386

**Published:** 2025-05-01

**Authors:** Rima Chaddad, Jamil Nasrallah, Waseem Sajjad, Eran Sim Wen Jun, Bharat Khialani

**Affiliations:** a Cardiology Department, Grand Hospital de l'Est Francilien, France; b General Medicine Department, Faculty of Medical Sciences, Lebanese University, Beirut, Lebanon; c Life Sciences Department, Faculty of Sciences, Lebanese University, Beirut, Lebanon; d King Edward Medical University, Mayo Hospital, Lahore, Pakistan; e Cardiology Departmentx, Faculty of Sciences, Singapore

**Keywords:** Hybrid rational Atherectomy, Intravascular lithotripsy, Angioplasty, Drug eluting stent, Percutaneous intervention, Impella

## Abstract

**Introduction:**

High-risk percutaneous coronary intervention (PCI) with mechanical circulatory support has emerged as an alternative to coronary artery bypass grafting (CABG) for patients with complex left main coronary artery disease who are at high surgical risk.

**Case presentation:**

A 69-year-old woman with diabetes mellitus, hypertension, hyperlipidemia, and a prior stroke presented with non-ST-segment elevation myocardial infarction. Echocardiography revealed a left ventricular ejection fraction of 25 %. Coronary angiography showed heavily calcified distal left main disease with critical ostial stenosis of the left anterior descending (LAD) and left circumflex (LCX) arteries. Deemed unsuitable for CABG, she underwent high-risk PCI with Impella mechanical support. Rotational atherectomy and intravascular lithotripsy were employed to address extensive calcification. A drug-coated balloon angioplasty was performed from the left main to the LCX, and a drug-eluting stent was placed from the left main into the mid-LAD. Post-dilation ensured optimal stent apposition and vessel patency.

**Discussion:**

Mechanical circulatory support provided essential hemodynamic stability during the complex PCI procedure. Advanced lesion modification techniques, including rotational atherectomy and intravascular lithotripsy, were crucial for treating the heavily calcified lesions, facilitating optimal stent deployment, and minimizing procedural risks.

**Conclusion:**

The hybrid PCI approach combining mechanical support and advanced interventional techniques was effective in managing complex coronary artery disease in a high-risk patient contraindicated for CABG. This strategy offers a viable alternative for patients with significant anatomical and clinical challenges, emphasizing the importance of tailored therapeutic interventions in contemporary interventional cardiology.

## Highlights

• Hybrid Rotational Atherectomy and Shockwave PCI Approach in a high-bleeding risk patient.
• Mechanical Circulatory Support in High-Risk PCI, the Impella CP device.
• Alternative to CABG in a High-Surgical-Risk Patient, the complex PCI.
• Optimized Stent Deployment with IVUS Guidance.
• This case highlights the evolving role of hybrid PCI strategies in managing complex coronary artery diseases.

## 1 Introduction

Coronary artery disease (CAD) remains a leading cause of morbidity and mortality worldwide, with acute presentations, such as non-ST-segment elevation myocardial infarction (NSTEMI), posing significant management challenges, especially in high-risk patients [1]. Traditionally, coronary artery bypass grafting (CABG) is the preferred revascularization strategy in cases of severe left main coronary artery disease with concomitant critical stenosis in major branches [2]. However, CABG may not be feasible for patients with advanced comorbidities and high surgical risks, necessitating alternative, less invasive approaches [3,4].

High-risk percutaneous coronary intervention (PCI) with mechanical circulatory support has emerged as a viable revascularization strategy for patients unable to tolerate CABG. This approach, often facilitated by devices such as the Impella, enables interventionalists to perform complex procedures with enhanced hemodynamic stability, which is particularly beneficial in individuals with severe left ventricular (LV) dysfunction and extensive coronary calcifications [5]. For patients with substantial calcific plaque burden, achieving adequate lesion modification is critical, as it improves stent expansion and long-term patency. Techniques such as rotational atherectomy and intravascular lithotripsy (IVL) have shown promise in effectively addressing these heavily calcified lesions, paving the way for successful drug-eluting stent (DES) placement [6].

This case report describes the management of a 69-year-old woman with significant comorbidities, including diabetes mellitus, hypertension, hyperlipidemia, and prior cerebrovascular accident, who presented with NSTEMI and severe distal left main disease complicated by ostial stenosis in both the left anterior descending (LAD) and left circumflex (LCX) arteries. Due to the patient's elevated surgical risk, a multidisciplinary team opted for a high-risk PCI strategy incorporating mechanical circulatory support and advanced lesion modification techniques to achieve optimal revascularization. This case highlights the clinical importance and utility of combining rotational atherectomy and IVL in heavily calcified coronary lesions, along with mechanical support, as an effective alternative in patients contraindicated for CABG.

This hybrid strategy, particularly essential for anatomically complex and high-risk patients, underscores the indispensable role of mechanical circulatory support—such as the Impella device—in maintaining hemodynamic stability and preventing lethal complications such as arrhythmias or cardiac arrest.

An implied consent was taken from the patient for publication of this case report and the accompanying images. The patient was made sure that any information identifying her will be kept confidential and this research will only be used for academic and educational purpose and for better patient outcomes in similar scenarios.

## 2 Case history/examination

**Fig. 1:**
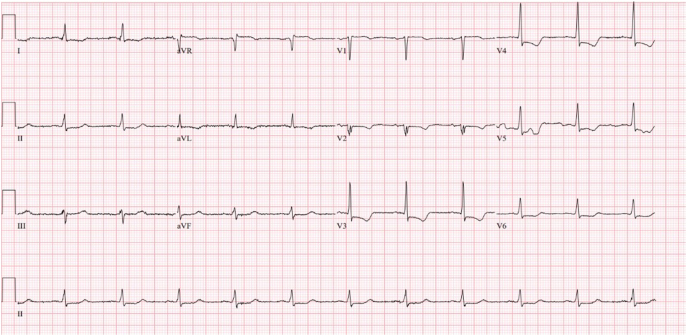
ECG showing NSTEMI.

A 69-year-old woman with a medical history of diabetes mellitus, hypertension, hyperlipidemia, and a prior stroke presented to the emergency department with chest pain and was diagnosed with NSTEMI (Fig. 1). Transthoracic echocardiography revealed a markedly reduced left ventricular ejection fraction of 25 %, indicating severe left ventricular dysfunction.

## 3 Differential diagnosis, investigations and treatment

**Fig. 2:**
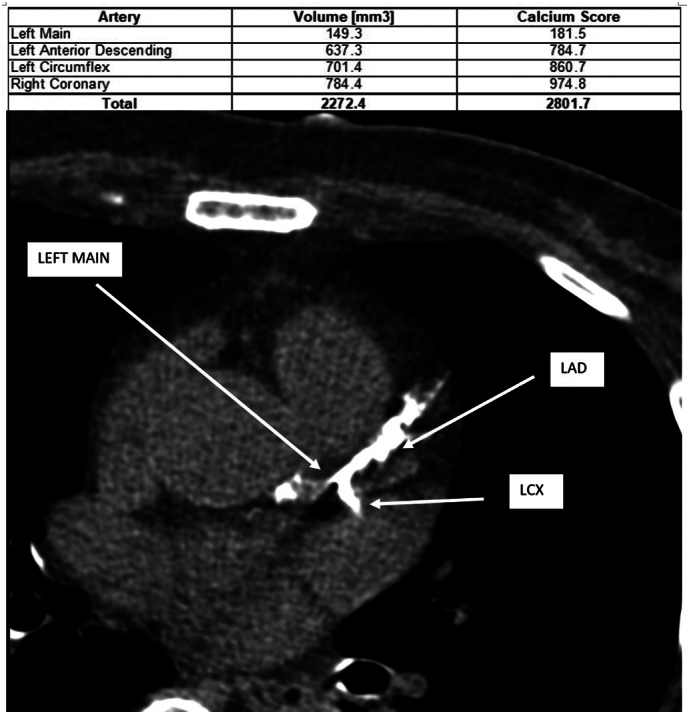
Coronary angiography showing heavy coronary calcifications and severe distal left main coronary artery disease, along with ostial stenosis of LAD and LCX arteries.

Urgent coronary angiography was performed, revealing heavily calcified and severe distal left main coronary artery disease, along with critical ostial stenosis of both the left anterior descending (LAD) and left circumflex (LCX) arteries (Fig. 2). An intra-aortic balloon pump (IABP) was initially placed to provide hemodynamic support. Given the patient's high surgical risk and contraindications for coronary artery bypass grafting (CABG), a multidisciplinary team—including interventional cardiologists and cardiothoracic surgeons—concluded that high-risk percutaneous coronary intervention (PCI) with mechanical support was the most appropriate course of action.

After informed consent was obtained, the IABP was removed, and vascular access was secured using an 8 French (Fr) sheath in the groin, followed by the deployment of two Perclose ProGlide closure devices. The sheath was then upsized to a 14 Fr peel-away sheath to accommodate the Impella CP mechanical circulatory support device. A 5 Fr angulated pigtail catheter was advanced into the left ventricle over a 0.035-in. guidewire, which was then exchanged for a 0.018-in. wire to facilitate the placement of the Impella device.

**Fig. 3:**
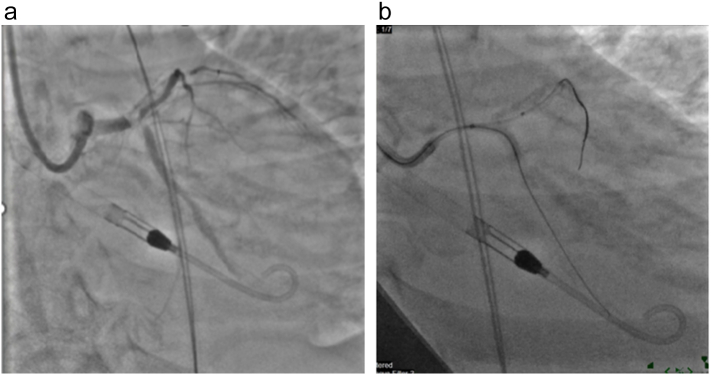
**a:** Impella implanted **b:** Impella implanted.

Coronary engagement was achieved using a 7 Fr EBU guide catheter. Wiring of the LAD and LCX was challenging due to the extensive calcification but was eventually successful (Figs. 3 **and 3b**).

**Fig. 4:**
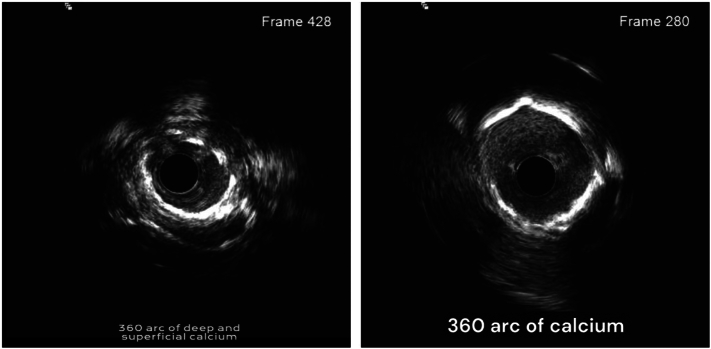
IVUS of LAD and Cx showing 360 degrees of calcifications.

Sequential balloon predilation of both arteries was performed using various balloon sizes. Intravascular ultrasound (IVUS) imaging demonstrated significant fibrocalcific plaque with deep and superficial calcium deposits in the affected vessels (Fig. 4).

**Fig. 5:**
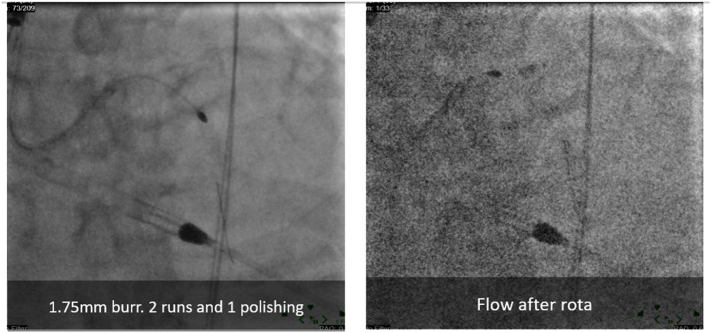
Rotational atherectomy was conducted on both the LCX and LAD to modify the plaque and facilitate stent delivery using Rotablator 1.75 mm.

Given the substantial calcific burden, rotational atherectomy was conducted on both the LCX and LAD to modify the plaque and facilitate stent delivery (Fig. 5). This was followed by intravascular lithotripsy (IVL) using Shockwave C2 balloons to further address the calcified lesions in the left main bifurcation into the LAD and LCX. A drug-coated balloon (DCB) angioplasty was then performed from the left main stem to the LCX to enhance luminal gain without additional stent placement.

Subsequently, a 3.5 × 32 mm Synergy Megatron drug-eluting stent (DES) was deployed from the proximal left main into the mid LAD at 12 atm, achieving optimal stent expansion. Post-dilation was carried out using a 4.25 mm non-compliant balloon to ensure proper stent apposition and vessel patency. Final angiographic images revealed satisfactory results with improved coronary flow (TIMI grade 3), although minor residual stenosis was noted at the ostial LCX. Given the adequate flow, no further intervention on the LCX was pursued.

## 4 Conclusion and results (outcome and follow-up)

**Fig. 6:**
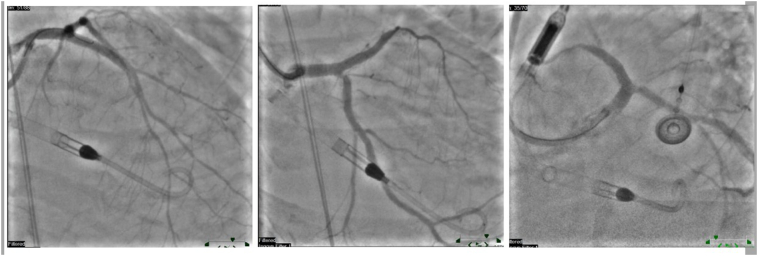
Final IVUS imaging confirmed favorable stent apposition and an acceptable luminal diameter in the treated segments.

**Fig. 7:**
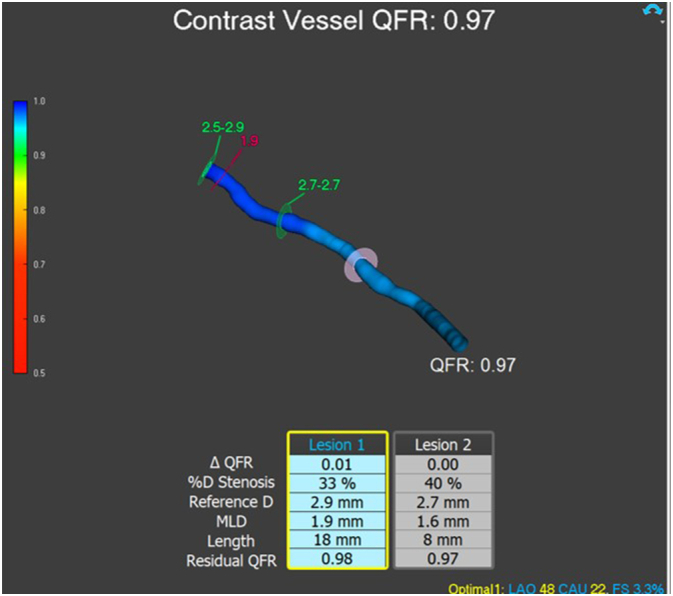
Quantitative Flow Ratio (QFR) Analysis of Coronary Artery Lesions: Evaluating Stenosis and Residual Flow, showed that the LCX artery after DCB angioplasty had good flow functionally.

Final IVUS imaging confirmed favorable stent apposition and an acceptable luminal diameter in the treated segments (Fig. 6). The QFR result (0.97) indicates that the identified coronary lesions impose minimal functional significance on blood flow, suggesting no hemodynamically relevant obstruction (Fig. 7). The Impella CP device was gradually weaned and successfully removed in the catheterization laboratory. Hemostasis at the access site was achieved using the previously placed ProGlide devices and a 6 Fr Angio-Seal closure device. Additional balloon tamponade was applied via the right radial artery approach to ensure vascular closure integrity. The patient remained hemodynamically stable throughout the procedure and was transferred to the intensive care unit for further monitoring and management.

## 5 Discussion

Cardiogenic shock following myocardial infarction (MI) is a life-threatening condition characterized by the heart's inability to pump sufficient blood to meet the body's needs, leading to inadequate tissue perfusion and potential organ failure [7]. This condition typically arises from extensive myocardial damage after an MI, resulting in severe impairment of left ventricular function. Clinically, cardiogenic shock is defined by sustained hypotension (systolic blood pressure < 90 mm Hg for at least 30 min), signs of hypoperfusion—such as altered mental status, cold and clammy skin, and oliguria—and elevated filling pressures indicated by a pulmonary capillary wedge pressure > 15 mm Hg [7]. Despite advances in reperfusion therapy and mechanical circulatory support devices, cardiogenic shock remains a major cause of mortality and morbidity among MI patients [8].

Managing complex PCI in the setting of cardiogenic shock presents significant therapeutic challenges due to the severe hemodynamic instability and high-risk coronary anatomy often encountered. Complex PCI procedures may involve multivessel disease, bifurcation lesions, or chronic total occlusions, requiring advanced techniques and expertise to achieve successful revascularization. The use of mechanical circulatory support devices, such as the Impella or intra-aortic balloon pump (IABP), provides critical hemodynamic support during these high-risk procedures by maintaining perfusion and reducing ventricular workload [9]. Early revascularization combined with mechanical support has been shown to enhance survival rates and promote myocardial recovery in patients with cardiogenic shock undergoing complex PCI [8]. Optimal management strategies necessitate individualized assessment and a multidisciplinary approach to address the unique challenges presented by each case.

The IABP plays a crucial role in the management of cardiogenic shock, particularly in patients with acute MI. By inflating during diastole and deflating just before systole, the IABP enhances coronary perfusion and decreases left ventricular afterload, thereby improving cardiac output and reducing myocardial oxygen demand [10]. Clinical studies have demonstrated that the IABP can stabilize hemodynamic parameters and provide temporary support to patients in cardiogenic shock, facilitating recovery or acting as a bridge to more definitive treatments such as revascularization or advanced mechanical circulatory support [8,11]. However, recent large-scale trials, such as the IABP-SHOCK II trial, have questioned its impact on long-term mortality, suggesting that its role should be carefully considered within the broader context of patient-specific factors and evolving therapeutic options [12].

The Impella device, a percutaneous ventricular assist device, has gained increasing significance in the management of cardiogenic shock, especially during urgent PCI. By providing continuous axial blood flow from the left ventricle to the aorta, the Impella reduces left ventricular workload, enhances cardiac output, and improves end-organ perfusion. This mechanical support allows for hemodynamic stabilization, creating a more controlled environment for urgent PCI—critical in patients with severe myocardial dysfunction or those undergoing complex coronary interventions [5]. Studies have shown that the use of Impella can lead to improved outcomes, such as increased survival rates and better recovery of myocardial function compared to conventional support methods [13]. Nonetheless, the benefits must be weighed against potential complications, including vascular injury and hemolysis, emphasizing the need for careful patient selection and management. Emerging data, such as from the ROTATE-IVL registry and the CALCIVES study, suggest that combining RA with IVL may result in better lesion compliance and procedural success compared to either modality alone [6,15,16]. This hybridization allows for improved deliverability and enhanced luminal gain in severely calcified lesions. Although the patient did not experience complications, it is important to acknowledge the procedural risks associated with these techniques [15,16]. Rotational atherectomy carries risks of slow-flow or no-reflow, vessel perforation, and distal embolization. IVL may be associated with balloon rupture, and its high cost may limit its accessibility in some settings. Recognizing these risks adds credibility to the procedural planning [6,15,16]. The rationale behind performing RA before IVL stems from the distinct mechanical properties of these modalities. Rotational atherectomy was used first to ablate superficial and nodular calcifications, enabling the passage of subsequent balloons. IVL, subsequently employed, effectively fractured deeper concentric calcium, optimizing vessel compliance. This sequence, RA followed by IVL, represents a complementary and synergistic approach to lesion preparation.

## 6 Conclusion

The successful management of a high-bleeding risk patient with complex left main coronary artery disease using a Rota-Shock hybrid PCI approach highlights the advanced capabilities and multidisciplinary coordination in contemporary interventional cardiology. The utilization of rotational atherectomy was crucial in modifying heavily calcified lesions, ensuring optimal stent deployment. Mechanical circulatory support devices, such as the Impella and intra-aortic balloon pump, provided essential hemodynamic stability throughout the procedure. This comprehensive strategy not only mitigated the risks associated with extensive coronary manipulation and potential hemodynamic compromise but also effectively addressed the bleeding concerns inherent in high-bleeding risk patients. The positive outcome in this case underscores the importance of tailored therapeutic strategies that combine cutting-edge interventional techniques with meticulous peri-procedural management to enhance safety and efficacy in high-risk populations. This hybrid approach represents a significant advancement in the treatment paradigm for complex coronary interventions, offering a viable and effective option for patients facing significant anatomical and clinical challenges. This case serves as an illustrative example of the progressive integration of advanced plaque modification strategies—namely, rotational atherectomy and intravascular lithotripsy—within a single high-risk PCI procedure. Rather than a first-of-its-kind case, it reflects a growing shift toward hybridized interventional strategies in contemporary practice.

This work has been reported in line with the SCARE criteria [14].

## Author contribution

All authors contributed to the manuscript equally.

## Consent statement

Written informed consent was obtained from the patient for publication of this case report and accompanying images.

## Ethical approval

Ethical approval was waived based on the observational nature of the report.

## Guarantor

Jamil Nasrallah.

## Funding

This research did not receive any specific grant from funding agencies in the public, commercial, or not-for-profit sectors.

## Declaration of competing interest

None.
